# Deterioration of Cement-Based Materials in Low-Temperature Seawater

**DOI:** 10.3390/ma16155278

**Published:** 2023-07-27

**Authors:** Mari Kobayashi, Keisuke Takahashi, Yuichiro Kawabata

**Affiliations:** 1 Mitsubishi UBE Cement Corporation, 1-6 Okinoyama, Yamaguchi 755-8633, Japan; keisuke.takahashi@mu-cc.com; 2 Port and Airport Research Institute, 3-1-1 Nagase, Kanagawa 239-0826, Japan; kawabata-y@p.mpat.go.jp

**Keywords:** deep sea, seawater attack, scanning electron microscopy–energy-dispersive X-ray spectroscopy, paste, thaumasite, ettringite

## Abstract

Cementitious materials have potential for infrastructure development in low-temperature marine environments, including in seawater at high latitudes and in deep-sea environments (water depths of >1000 m). Although the marine deterioration of cementitious materials has been widely investigated, the influence of seawater temperature has not been elucidated. In this study, to determine the effects of low-temperature seawater on the durability of cementitious materials, cement paste specimens were immersed in a seawater tank at room temperature and 2 °C for 433 days. The specimen immersed in low-temperature seawater exhibited significant deterioration with a partially collapsed surface, whereas the specimen immersed in room-temperature seawater maintained its original shape. Following low-temperature immersion, Ca dissolution was more pronounced and dissolved portlandite, decalcified calcium (alumino)silicate hydrate (C–(A-)S–H), magnesium (alumino)silicate hydrate (M–(A-)S–H), and thaumasite were observed on the collapsed surface. Such significant deterioration can be attributed to the increased solubility of portlandite under low-temperature conditions, which could promote Ca dissolution and subsequently lead to C–(A-)S–H decalcification and the formation of M–(A-)S–H and thaumasite. These insights are expected to contribute to the successful construction and maintenance of cementitious structures in low-temperature seawater.

## 1. Introduction

The marine deterioration of cementitious materials has been studied extensively [1,2,3,4,5,6,7,8,9,10,11] because the variety of potentially aggressive ions present in seawater results in marine concrete structures having much shorter lifetimes than land-based structures [12]. Numerous ions are present in seawater, including chloride (Cl^−^), sodium (Na^+^), magnesium (Mg^2+^), sulfate (SO_4_^2−^), calcium (Ca^2+^), and carbonate and bicarbonate ions (CO_3_^2−^ and HCO_3_^−^). The ingress of Cl^−^ into reinforced concrete structures can cause severe cracks, resulting in the corrosion of steel reinforcements and a short service life [13]. In addition, the ion concentration gradient at the interface between concrete and seawater can act as a driving force for diffusion, causing the ions in seawater to diffuse into the concrete, whereas Ca^2+^ and hydroxide ions (OH^−^) in the concrete are leached out. The leaching of Ca^2+^ and OH^−^ leads to damage via the dissolution of portlandite and the decalcification of calcium (alumino)silicate hydrate (C–(A-)S–H), which are the main components of cement hydrates. Moreover, the ions originating from seawater can interact with the cement hydrates to form noncementitious hydrates, resulting in gradual degradation.

Upon diffusion into hardened cement specimens, Cl^−^ can substitute SO_4_^2−^ in the monosulfate to form Friedel’s salt (3CaO·Al_2_O_3_·CaCl_2_·10H_2_O) or Kuzel’s salt (3CaO·Al_2_O_3_·0.5CaCl_2_·0.5CaSO_4_·10H_2_O) [14,15,16]. Cl^−^ can also be adsorbed on C–(A-)S–H [17,18,19]. These processes are believed to be harmful to binders.

Following ingress, Mg^2+^ from seawater can react with OH^−^ in the pore solution (pH > 9.4) to form brucite (Mg(OH)_2_) [2], which consumes OH^−^ and lowers the alkalinity of the pore solution, thereby promoting the dissolution of portlandite and decalcification of the C–(A-)S–H phase [20,21]. When the brucite phase becomes undersaturated at lower pH values, Mg^2+^ can precipitate by reacting with decalcified C–S–H or amorphous silica derived from Ca-deficient C–S–H [22,23], leading to the formation of magnesium silicate hydrate (M–S–H). The M–S–H phase has a weak talc-like structure, which can cause the collapse of specimen surfaces [24,25].

SO_4_^2−^ from seawater can form ettringite (3CaO·Al_2_O_3_·3CaSO_4_·32H_2_O) and gypsum (CaSO_4_·2H_2_O). However, owing to the low SO_4_^2−^ concentration in seawater, no or negligible gypsum formation occurs [11,26]. Furthermore, ettringite formation does not typically lead to serious SO_4_^2−^ attack on concrete exposed to seawater. Even with SO_4_^2−^ enrichment and ettringite formation, a lack of damage, such as cracking and spalling, has been reported [9,27]. Degradation resulting from ettringite formation during SO_4_^2−^ attack is generally ascribed to the crystallization pressure from a supersaturated solution [28,29]. Although the reason for the lack of deterioration in the sulfur-rich zone remains unclear, other ions in seawater may affect the solubility or supersaturation of ettringite [9].

HCO_3_^−^ and CO_3_^2−^ from seawater can react with Ca^2+^ ions in the pore solution to form calcium carbonate. At temperatures below 15 °C, the presence of reactive silica derived from decalcified C–(A-)S–H, SO_4_^2−^, and HCO_3_^−^ and CO_3_^2−^ induce the formation of thaumasite (CaSiO_3_·CaCO_3_·CaSO_4_·15H_2_O) [8,30]. This process transforms the matrix into a soft mass without any binding capacity [30].

The durability of cementitious materials has been investigated in various marine environments, as exemplified in Table 1. Previous studies have been conducted in shallow sea areas or laboratories, and most provided no information on the temperature of the test area. Table 1 lists the minimum and maximum seawater temperatures near the test area, as obtained from a database [31]. Although certain studies have investigated the deterioration of concrete under harsh testing conditions [1,7,9,10], the durability of cementitious materials has not yet been measured at constant low temperature. Moreover, the property changes measured in these studies were combined with those measured for freeze-thaw deterioration. The temperature of the surface layer varies naturally with the season, even in the same area, and relatively low temperatures occur at high latitudes. As temperature influences the diffusion of ions from seawater into concrete, the durability of concrete structures is expected to be affected by temperature. Furthermore, the stability and solubility of the hydrates constituting cementitious materials are expected to depend on temperature, which could also affect durability. Reports on sulfate attack have suggested that low temperatures can accelerate deterioration. Kobayashi et al. and Takahashi et al., who investigated the durability of cement mortars under deep-sea conditions at 2–4 °C, found that that deterioration of cementitious materials was significantly promoted [32,33]. Although the mechanisms of severe deterioration are not fully understood, low temperatures may play an important role. However, no studies have focused on the effects of low temperatures on the marine degradation of cementitious materials. An improved understanding of and experimental data related to the influence of low-temperature seawater on the durability of cementitious materials would facilitate the successful construction and subsequent maintenance of cement-based concrete structures under very low-temperature conditions, such as in deep seas and Antarctica.

In this study, the effects of low-temperature seawater on the durability of cementitious materials were investigated. Cement paste specimens were immersed in water tanks controlled at 2 °C or room temperature for 433 days. After immersion, changes in the hydrate phases of the specimens were analyzed using X-ray diffraction (XRD), scanning electron microscopy–energy-dispersive X-ray spectroscopy (SEM-EDS), ^29^Si nuclear magnetic resonance (NMR) spectroscopy, and electron probe microanalysis (EPMA). In addition, the differences in the durability of cementitious materials due to varying seawater temperatures are discussed.

## 2. Experimental Method

### 2.1. Materials and Sample Preparation

Portland cement (PC) was used for the paste specimens. The chemical composition of the PC was determined by X-ray fluorescence (XRF) (Simultix 14, Rigaku Corporation, Tokyo, Japan) analysis according to the JIS R 5204 standard [34] (Table 2). The paste mix was originally designed for antiwashout underwater concrete. The mix proportions of the paste specimens are listed in Table 3. The paste had a water-to-cement ratio of 0.6 and contained a polycarboxylic ether (PCE) superplasticizer (BASF Construction Additives GmbH, Trostberg, Germany), hydroxypropyl methylcellulose (HPMC) thickener (Shin-Etsu Chemical Co., Ltd., Tokyo, Japan), and defoamer (ADEKA CORPORATION, Tokyo, Japan). The paste was mixed using a mechanical mixer at 700 rpm for 2 min and cast into a 40 × 40 × 160 mm^3^ mold. The specimens were then sealed and cured at 20 °C for 28 days before seawater immersion.

### 2.2. Laboratory Immersion Tests

Two water tanks with volumes of 216 L were prepared for specimen immersion in room-temperature seawater and in low-temperature seawater. Room-temperature seawater consisted of seawater in a laboratory where the room temperature was not precisely controlled. The temperature of the seawater was monitored over 1 year, and the maximum and minimum recorded temperatures were 27 and 10 °C, respectively. The temperature of the low-temperature seawater was controlled at 2 ± 1 °C using a thermocontroller. Fresh seawater from the Kurihama Bay was supplied to each tank at a flow rate of 150 mL/min, and the seawater in the tank was replaced approximately every 24 h. The specimens were immersed in the tanks for 433 days.

### 2.3. Characterization Methods

**Figure 1:**
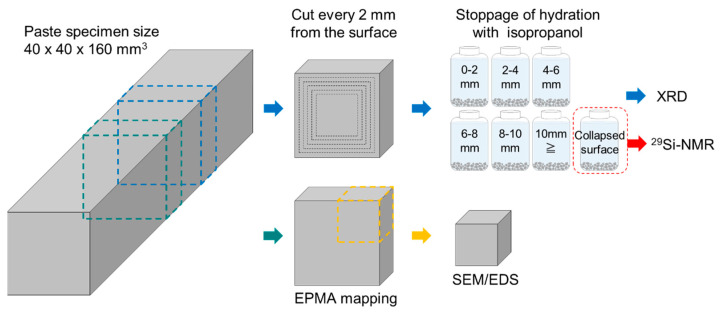
Preparation of paste specimens for analysis following seawater immersion. The dotted lines indicate the cutting planes.

After the immersion test, each specimen was sliced into two pieces with a thickness of 10 mm using a table bench saw with liquid paraffin, as shown in Figure 1. For EPMA, one sample was impregnated with epoxy resin, polished, and coated with carbon. After EPMA, the sample was cut into 20 × 20 × 10 mm^3^ pieces and polished again for SEM-EDS analysis. The other sample was cut using a low-speed cutter (IsoMet Cutter, Buhler, Lake Bluff, IL, USA) into 2 mm slices from the surface to a depth of 10 mm, and the remaining sample was denoted as the bulk sample. Mashy collapsed areas on the surface of the specimen immersed in low-temperature seawater were collected directly and denoted as the collapsed surface sample. Each piece was crushed to pass through a 5 mm sieve and treated with isopropanol to stop hydration. For the dehydration process, the samples were immersed in isopropanol and the solvent was changed after 6 h and again after 24 h. Then, after immersion for 7 days, the samples were dried in a thermostatic bath at 35 °C for 1 h. Finally, the samples were ground into a powder of less than 90 μm.

#### 2.3.1. XRD Analysis

XRD patterns were collected using a D2 PHASER diffractometer (Bruker AXS GmbH, Karlsruhe, Germany) in a θ–2θ configuration with Cu-Kα radiation (λ = 1.54 Å) at a voltage of 30 kV and current of 10 mA with steps of 0.02° and a speed of 2.4°/min in the 2θ range of 5–70°. Phase identification was performed using the TOPAS software (DIFFRAC. TOPAS, Version 5).

#### 2.3.2. EPMA

EPMA allows for the quantitative analysis of the atomic distribution in a cross-sectional area [35]. Elemental mapping was obtained based on the characteristic X-ray intensity measured for each element, where the mass percentages were converted using the ZAF correction. The EPMA samples consisted of 10 mm thick slices, as shown in Figure 1, which were dried for 2 weeks under vacuum conditions and then embedded in epoxy resin. After the epoxy resin hardened, the surface was dry polished using #120–#4000 grit SiC grinding paper. The polished surface was then sputter coated with a conductive carbon layer.

EPMA was performed using a JXA-8200 instrument (JEOL, Ltd., Tokyo, Japan). SO_3_, Cl, MgO, Al_2_O_3_, and CaO were measured using a wavelength-dispersive spectrometer with an acceleration voltage of 15 kV, probe diameter of 50 µm, unit measurement time of 40 ms/point, pixel size of 100 µm, and beam current of 200 nA.

#### 2.3.3. SEM-EDS Analysis

SEM-EDS measurements were performed using a scanning electron microscope equipped with a backscattering electron detector and an X-ray energy-dispersive spectrometer (JSM-IT-300, JEOL, Ltd., Tokyo, Japan). An accelerating voltage of 15 kV and working distance of 10.0 mm were used. A high-resolution set of backscattered electron (BSE) images was acquired to analyze the macro–micro information. An SEM montage was constructed by electronically stitching together 92 BSE images obtained at a magnification of 200×. The area and amount of microcracks in the sections of the SEM montage were calculated using the ImageJ software (Version v1.54d, National Institutes of Health, Bethesda, MD, USA), and the result of this calculation was defined as the porosity.

For EDS measurements, a volume of paste comprising a mixture of phases was examined while avoiding cracks and aggregates. The EDS point analysis results are presented as atomic ratios in dot plots and are compared with the theoretical compositions of typical hydrate and mineral phases that were considered to form in the paste (Appendix A and Appendix B). The mixture of phases in the paste volume observed by EDS analysis falls within the theoretical composition of these phases.

#### 2.3.4. Magic-Angle Spinning (MAS) NMR Spectroscopy

Solid-state ^29^Si MAS NMR measurements were performed on an ECA 400 spectrometer (magnetic field: 9.2 T, JEOL Resonance) using the powdered collapsed surface sample employed for XRD analysis. Before loading, the sample was packed in a 4.0 mm zirconia rotor. ^29^Si MAS NMR spectra were acquired by collecting more than 3360 scans at a spinning speed of 16 kHz, 90° pulse duration of 3.8 μs, and relaxation delay of 40 s without ^1^H decoupling. The ^29^Si MAS NMR chemical shifts were referenced to those of an external tetramethylsilane (TMS) sample (δ^29^Si = 0.0 ppm). The ^29^Si NMR signals were analyzed using the Q^n^ classification, in which a Si tetrahedron is connected to n Si tetrahedrons with n varying from 0 to 4.

## 3. Results

### 3.1. Visual Changes

**Figure 2:**
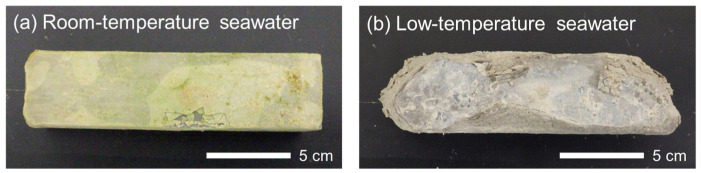
Paste specimens after immersion in (**a**) room-temperature seawater and (**b**) low-temperature seawater for 433 days.

Figure 2 shows the paste specimens after immersion in seawater in the laboratory for 433 days. The specimen immersed in room-temperature seawater was not significantly damaged, although the surface was covered with white precipitates of brucite. In contrast, the specimen immersed in low-temperature seawater exhibited significant surface deterioration. The specimen was partially collapsed, did not maintain its original shape, and had a fragile and mashy structure.

### 3.2. Elemental Mapping by EPMA

**Figure 3:**
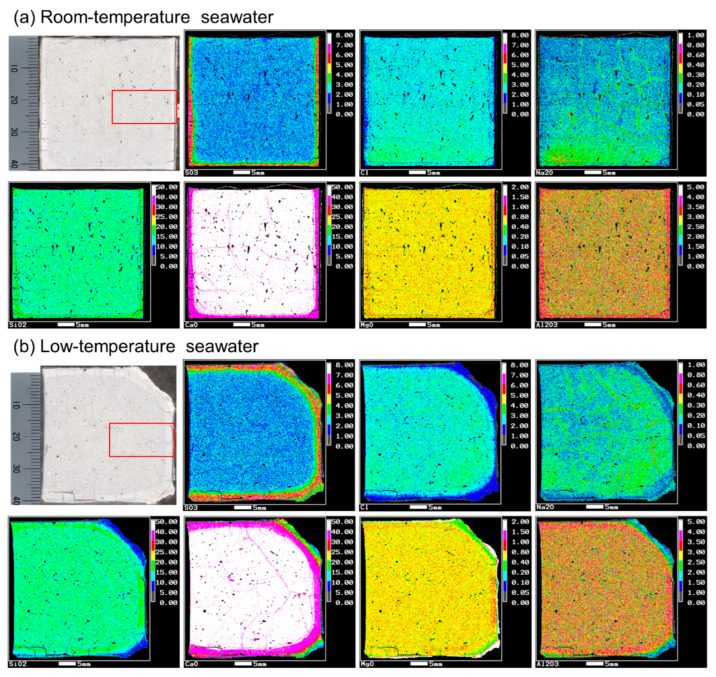
EPMA elemental maps of the PC paste specimens immersed in (**a**) room-temperature seawater and (**b**) low-temperature seawater. The area enclosed by the red square in the cross-sectional photographs indicate the area where the concentration profile analysis was performed for each element.

Elemental maps of each specimen were obtained to analyze ion diffusion from seawater and the associated dissolution of ions from the cement hydrate (Figure 3). For the specimen immersed in room-temperature seawater, changes in the chemical composition only occurred on the surface. However, the specimen immersed in low-temperature seawater showed changes in the chemical composition at greater depths. For example, Ca dissolution and SO_4_^2−^ diffusion fronts were present inside the low-temperature specimen. In addition, the corner area where the specimen collapsed had significantly lower Ca concentrations and higher Mg concentrations. This area of high Mg concentrations suggests the formation of a Mg phase, which is discussed in detail based on the EDS results. Overall, elemental mapping by EPMA revealed that specimen degradation was promoted by immersion in low-temperature seawater.

**Figure 4:**
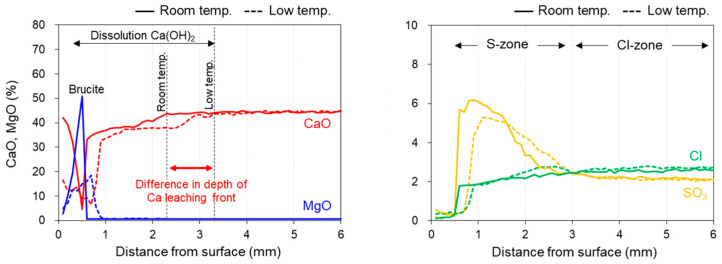
Concentration profiles of elements in the specimens immersed in room- and low-temperature seawater plotted from the surface (0 mm) to a depth of 6 mm within the area enclosed by the red square in Figure 3.

**Figure 5:**
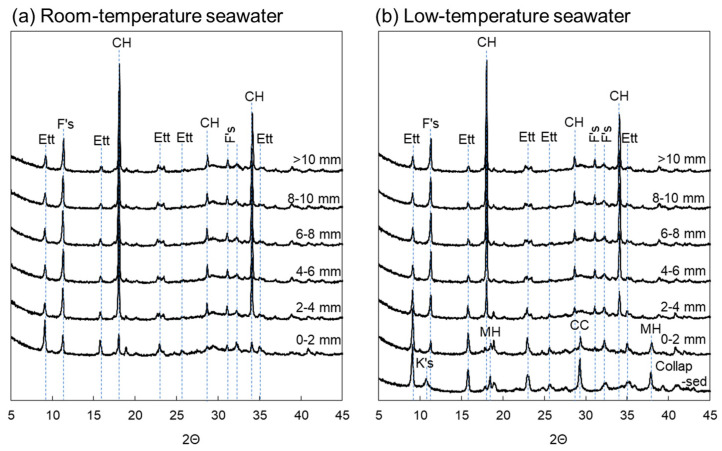
XRD patterns of the specimens obtained after immersion in (**a**) room-temperature seawater and (**b**) low-temperature seawater. Ett: ettringite, F’s: Friedel’s salt, K’s: Kuzel’s salt, CH: portlandite, CC: calcite, MH: brucite.

The concentration profile of each element was analyzed from the surface (0 mm) to the interior (20 mm) within the area enclosed by the red square in the cross-sectional photographs in Figure 3a,b. The profiles from the surface to a depth of 6 mm are shown in Figure 4. The concentrations were averaged every 100 µm. These results in combination with the XRD results at each depth (Figure 5) allow predictions of the types of hydrate that form or dissolve to induce changes in the element concentrations.

### 3.3. XRD Analysis

The Ca dissolution fronts were located at approximately 2 and 3 mm for the samples immersed in room-temperature and low-temperature seawater, respectively (Figure 4). The XRD patterns of the sample immersed in room-temperature seawater at a depth of 0–2 mm and those of the sample immersed in low-temperature seawater depths of 0–2 and 2–4 mm, which correspond to the Ca leaching area, exhibited very small portlandite peaks. This result indicates that portlandite was dissolved and smaller amounts of portlandite remained in these areas. Thus, portlandite dissolution was enhanced by immersing the specimens in low-temperature seawater. As shown in Figure 4, SO_4_^2−^ diffused further into the interior of the specimen immersed in low-temperature seawater compared with the specimen immersed in room-temperature seawater. The XRD patterns in Figure 5 show pronounced ettringite peaks at a depth of 0–2 mm for the specimen immersed in room-temperature seawater. The specimen immersed in low-temperature seawater also shows a prominent ettringite peak at 0–2 mm, and the peak intensity at 2–4 mm is higher than that further within the specimen (depth > 4 mm), indicating that ettringite formation in this area could involve SO_4_^2−^ from seawater. The XRD results indicate that ettringite also formed inside the specimen. Based on the EPMA results (Figure 3), Cl^−^ diffused into the center of the specimens during both room- and low-temperature seawater immersion because of its relatively high diffusion coefficient [36]. Consequently, Friedel’s salt was formed, as shown in Figure 5. Upon the formation of Friedel’s salt from the monosulfate by Cl^−^ substitution, SO_4_^2−^ is released into the solution in the pores, which promotes the simultaneous formation of ettringite [37]. Thaumasite could also form in the collapsed surface sample following immersion in low-temperature seawater. It has been reported that thaumasite forms in mortar specimens exposed to deep-sea conditions [32]. However, it is difficult to confirm the formation of thaumasite using XRD because of the similar structures of thaumasite and ettringite, both of which exhibit characteristic peaks at 2θ = 9°, 16°, and 23° [35,38]. Therefore, the possibility of thaumasite formation is discussed based on the ^29^Si NMR results.

Although the degree of deterioration varied with seawater temperature, the trends observed for ion diffusion into and out of the specimens were consistent with those previously reported for marine degradation [7,9,11,26]. Additionally, the trends identified by XRD for the crystalline hydrate phases at each depth agreed with the EDS point analysis results (Appendix B). However, the types of hydrates formed in fragile microstructures on the collapsed surfaces of the specimen immersed in low-temperature seawater could not be confirmed using XRD. Previous reports suggest that C–(A-)S–H decalcification as well as M–S–H and thaumasite formation can occur [26,30,32]. Accordingly, SEM-EDS was used to investigate the type of hydrate phase formed in the fragile zone at the surface.

### 3.4. SEM Imaging

**Figure 6:**
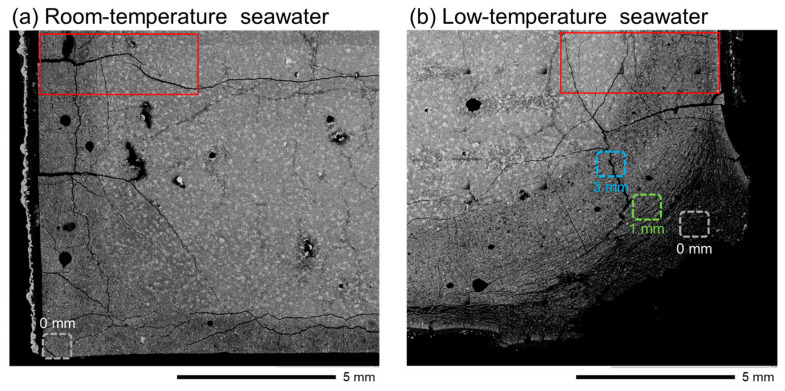
SEM montage images of the specimens after immersion in (**a**) room-temperature seawater and (**b**) low-temperature seawater. The areas surrounded by the red box indicate the area whose porosity (area %) was calculated by image processing.

**Figure 7:**
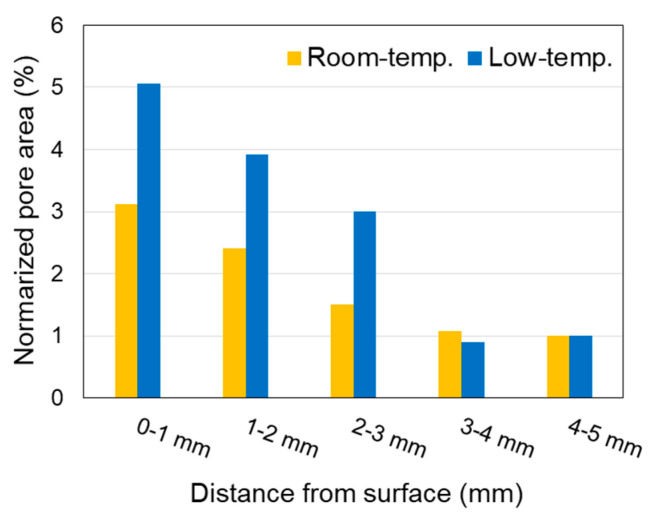
Porosity (area %) of the specimens immersed in room- and low-temperature seawater.

The SEM montages (Figure 6) reveal a grayscale change from the surface to the interior of each sample owing to hydrate dissolution. Each SEM montage image was analyzed in 1 mm sections from the surface to the interior of the specimen in the area surrounded by the red box, and the porosity (area %) was calculated by image processing (Figure 7). The threshold for binary images was set to 2 (min.) and 50 (max.) to exclude large cracks generated during sample preparation and entrapped/entrained pores. The porosity of each area was normalized to that at a depth of 4–5 mm. This analysis reveals that more pores existed close to the surface in both specimens. Compared with the specimen immersed in room-temperature seawater, that immersed in low-temperature seawater tended to have more pores and microcracks at the same depth, indicating that hydrate dissolution was more pronounced. Based on the EPMA and XRD results, the dissolved hydrate was mainly portlandite (Figure 3 and Figure 5).

EPMA revealed remarkable Ca dissolution at the collapsed corner of the specimen immersed in low-temperature seawater, similar to the behavior observed in a previous study under deep-sea conditions [32], and new hydrates formed on most of the collapsed surface. In particular, the EPMA results revealed a high Mg concentration in the area marked as 0 mm in Figure 6b, indicating the formation of Mg-based hydrates. To further understand the hydrates formed in this area, EDS point analysis was performed. For the specimen immersed in low-temperature seawater, the hydrate phases were evaluated based on the EDS point analysis results at depths of 1 mm, where fine parallel cracks occurred from the surface to the interior of the specimen, and 3 mm (see Appendix A for detailed SEM images).

### 3.5. EDS Analysis

**Figure 8:**
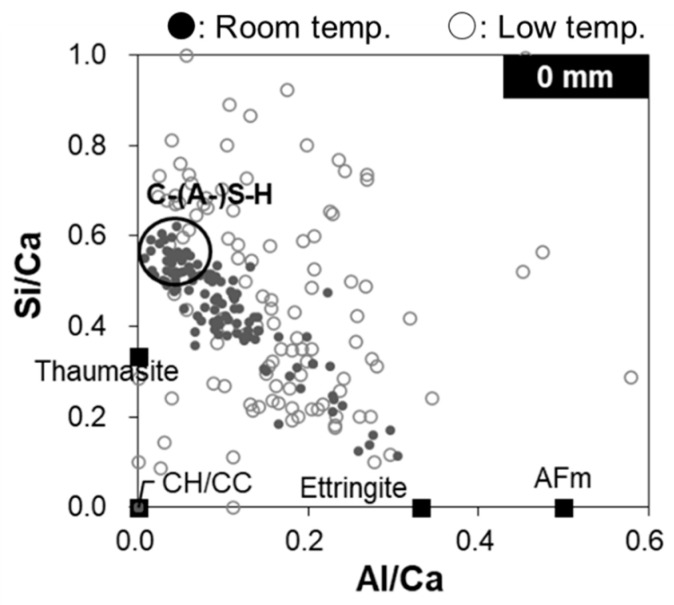
Si/Ca ratio as a function of the Al/Ca ratio at a depth of 0 mm for the specimens immersed in room- and low-temperature seawater.

Figure 8 shows the Si/Ca ratio as a function of the Al/Ca ratio near the specimen surface (0 mm depth), as determined by EDS point analysis of the specimens immersed in room- and low-temperature seawater. The circle denotes the typical C–(A-)S–H composition of ordinary PC, which has been widely reported in the literature [7,39,40]. The results for the specimen immersed in room-temperature seawater suggest that portlandite was dissolved, as the absence of data points near the origin indicates the presence of portlandite/calcium carbonate. The data points are not scattered in the circled area corresponding to a typical C–(A-)S–H composition, which indicates that C–(A-)S–H has not yet been decalcified. In addition, the data points lie on a line toward the theoretical composition of ettringite, which indicates the presence of ettringite formed by SO_4_^2−^ derived from seawater. In contrast, for the specimen immersed in low-temperature seawater, the data points are quite scattered and are located outside the circle corresponding to a typical C–(A-)S–H composition, suggesting the occurrence of C–(A-)S–H decalcification or the precipitation of new hydrates, such as Mg-based hydrates. Furthermore, in combination with the XRD results (Figure 5), the presence of some data points near the origin indicates that calcite is precipitated instead of portlandite.

**Figure 9:**
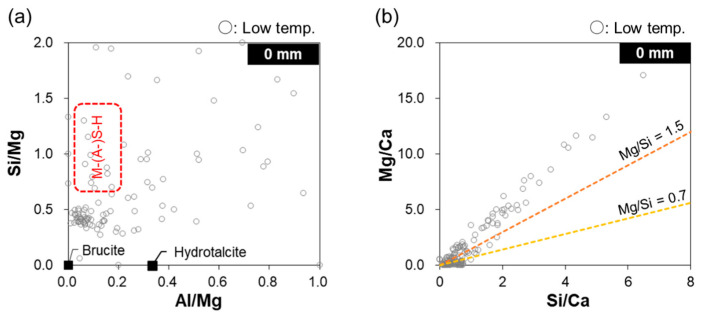
(**a**) Si/Mg ratio as a function of the Al/Mg ratio and (**b**) Mg/Ca ratio as a function of the Si/Ca ratio at a depth of 0 mm for the specimen immersed in low-temperature seawater.

Figure 9a shows the relative Si and Al atomic ratios with respect to the Mg content at a depth of 0 mm. The area enclosed by the red dotted line indicates the range of possible M–S–H and magnesium aluminosilicate hydrate (M–A–S–H) compositions [41]. Some data points are located in this area, indicating the formation of M–S–H or M–A–S–H. However, a cluster also occurs outside this area, near Si/Mg = 0.5, suggesting the intermixing of M–(A-)S–H and other Mg phases (likely brucite based on the XRD results). Figure 9b shows the Mg/Ca ratio as a function of the Si/Ca ratio at a depth of 0 mm. The data points form a line because M–S–H has a constant Mg/Si ratio. The two dotted lines in Figure 9b represent the lower and upper limits of the Mg/Si ratio for synthesized M–S–H [20]. As the observed data points are located above the upper limit of Mg/Si = 1.5, the precipitated M–(A-)S–H was likely intermixed with brucite.

Upon Mg^2+^ ingress into a cement paste specimen, it reacts with OH^−^ in the pore solution at pH > 9.5, forming brucite [2]. As this reaction consumes OH^−^, the pH of the pore solution decreases, which promotes further portlandite dissolution and eventually leads to the decalcification of C–(A-)S–H. At lower pH values, brucite becomes undersaturated and Mg^2+^ reacts with decalcified C–(A-)S–H to form M–S–H [9,11,26], which is stable at pH 7.5 [42]. However, as M–S–H possesses a weak talc-like structure [24,25], its formation causes surface collapse.

**Figure 10:**
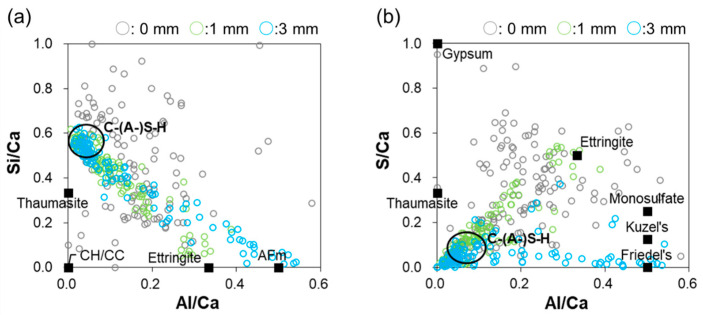
(**a**) Si/Ca ratio as a function of the Al/Ca ratio and (**b**) S/Ca ratio as a function of the Al/Ca ratio at depths of 0, 1, and 3 mm for the specimen immersed in low-temperature seawater.

Figure 10a shows plots of the Si/Ca ratio as a function of the Al/Ca ratio at depths of 0, 1, and 3 mm for the specimen immersed in low-temperature seawater. Portlandite was dissolved at both 1 and 3 mm because no data points appeared near the origin, and the points near the origin at a depth of 0 mm correspond to the formation of calcite, as discussed above. At a depth of 0 mm, the decalcification of C–(A-)S–H is observed, whereas at depths of 1 and 3 mm, many data points are located in the C–(A-)S–H circle, indicating that C–(A-)S–H decalcification could not occur. At a depth of 1 mm, the data points lie on a line from the C–(A-)S–H cluster toward the ideal composition of ettringite. At a depth of 3 mm, the data points lie on a line toward the AFm phase, which indicates that C–(A-)S–H and ettringite were present at a depth of 1 mm, whereas AFm was present at a depth of 3 mm. Figure 10b shows the plots of the S/Ca ratio as a function of the Al/Ca ratio at depths of 0, 1, and 3 mm for the specimen immersed in low-temperature seawater. At a depth of 3 mm, the data points lie on a line from C–(A-)S–H to the ideal composition of Friedel’s salt, indicating that the AFm phase in Figure 10a is predominantly Friedel’s salt.

### 3.6. ^29^Si-NMR Analysis

**Figure 11:**
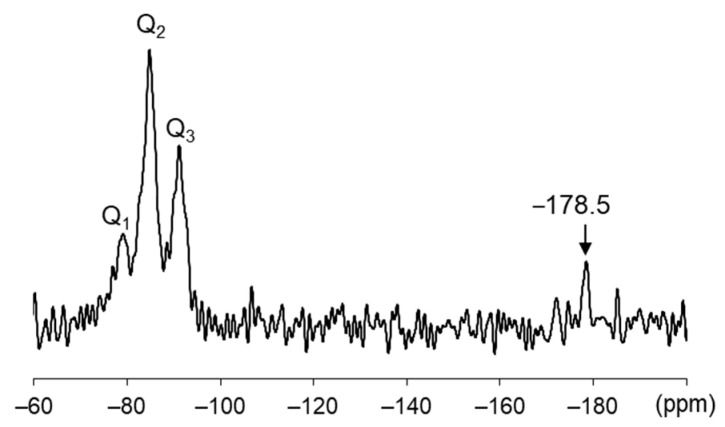
^29^Si MAS-NMR spectrum of the collapsed surface sample from the specimen immersed in low-temperature seawater.

Figure 11 shows the ^29^Si MAS NMR spectrum of the collapsed surface sample for the specimen immersed in low-temperature seawater. Richardson and Andersen et al. reported that the ^29^Si MAS NMR spectra of C–S–H exhibit two resonances originating from the Si end of the Q^1^ chain between −78 and −81 ppm and the bridging pair of Si tetrahedra in the dreierketten Si chain structure (Q^2^) between −82 and −86 ppm [39,43,44]. Thus, the intense peak at −84.8 ppm was assigned to the Q^2^ resonance, and the small peak at −79.1 ppm was assigned to the Q^1^ resonance (Figure 11). The decalcification of C–(A-)S–H strongly affects the balance between the Q^2^ and Q^1^ resonances, as Ca^2+^ leaching increases the length of the Si chain and thus the Q^2^/Q^1^ ratio [45]. Furthermore, Ca^2+^ leaching promotes the three-dimensional bonding of Si tetrahedra with other silicate anions, leading to a small and broad Q^3^ resonance [21]. The Q^3^ resonance at −91.2 ppm (Figure 11) also indicates the formation of M–(A-)S–H [24,46,47,48]. Bernard et al. recently reported that the ^29^Si MAS NMR spectrum of synthesized M–(A-)S–H exhibits intense and sharp Q^3^ resonances between −92 and −97 ppm [41]. The ^29^Si-NMR results for the collapsed surface sample support the decalcification of C–(A-)S–H and M–(A-)S–H formation, as revealed by EDS.

The detection of Si atoms with octahedral coordination at −178.5 ppm indicates the formation of thaumasite, which is the only hydrate phase in cementitious systems that contains Si atoms with this coordination [49]. SiO_6_ groups exhibit a chemical shift between −170 and −220 ppm, and thaumasite exhibits a single resonance at −179.4 ± 0.1 ppm in the ^29^Si MAS NMR spectrum [49].

## 4. Discussion

The specimen immersed in low-temperature seawater showed more significant degradation than that immersed in room-temperature seawater. Temperature has been reported to affect the durability of cementitious materials; for example, SO_4_^2−^ attack is accelerated at low temperatures, which is often attributed to the increased solubility of portlandite at low temperatures and the weakening of the surface layer owing to thaumasite formation [50]. The solubility of portlandite in cement increases at lower temperatures [51]. The saturation concentration of Ca^2+^ in portlandite is 1.08 times higher at 2 °C than at 20 °C [52], and more portlandite can dissolve in low-temperature environments. A steeper Ca^2+^ concentration gradient between seawater and the pore solution could drive further Ca dissolution (Figure 3). As portlandite dissolves, the porosity increases, as shown in Figure 7. This behavior could promote the diffusion of ions in seawater into the specimen and increase portlandite dissolution, resulting in the specimen immersed in low-temperature seawater exhibiting a greater degree of deterioration than the specimen immersed in room-temperature seawater.

Although portlandite dissolution increased the porosity of the specimen, as shown in Figure 7, it did not result in a mashy structure or surface collapse. For the specimen exposed to room-temperature seawater, no other damage was observed despite the dissolution of portlandite in the surface layer. However, portlandite dissolution increases seawater permeability, thereby facilitating C–(A-)S–H decalcification and finally the formation of M–(A-)S–H and thaumasite. The formation of these hydrates is expected to cause the surface layer to collapse.

Furthermore, damage to the specimen near the surface can be caused by ettringite formation. A sulfur-rich zone was observed near the specimen surfaces owing to the formation of ettringite. In the specimen immersed in low-temperature seawater, this zone was located at a depth of approximately 1 mm, and fine parallel cracks appeared around this area (Figure 6), suggesting that the specimen began to collapse in this area. However, ettringite does not always lead to serious SO_4_^2−^ attack in concrete, with no cracking or spalling observed in sulfur-rich zones [9,53]. In addition, ettringite formation can generate expansive pressure. In particular, ettringite precipitation in smaller pores (typically < 20 nm) in the hydrated matrix can cause specimen expansion [54], and the pores must be supersaturated with respect to ettringite [54]. The solubility of ettringite decreases at lower temperatures [55], and its precipitation becomes more pronounced. As a result, ettringite formation may produce higher expansion pressure in a specimen immersed in low-temperature seawater than that immersed in room temperature seawater, which may damage the specimen. In addition, ettringite formation may exert expansion pressure at the area where SO_4_^2−^ penetrates the specimen from seawater; this expansion pressure may then act toward the relatively less constrained surface rather than the less damaged interior of the specimen, resulting in cracks parallel to the surface.

Is it contradictory to suggest that the formation of ettringite in a small pore could cause an expansion pressure in a saturation where the porosity is increasing due to the dissolution of portlandite? Maybe not. Portlandite is usually crystalline in nature, and its crystals are above a few microns in size. The SEM-EDS result at the depth of 1 mm (Figure 6 and Figure 10) suggest that C–(A-)S–H was not decalcified and appeared to be mixed with ettringite even after the dissolution of portlandite. Moreover, C–(A-)S–H may contain small pores such as gel pores (~10 nm [56]), and ettringite formation in such pores may produce expansion pressure [54]. Therefore, although ettringite formation in low-temperature seawater may have contributed to the collapse of the specimen, the deterioration of the specimen needs to be investigated further.

## 5. Conclusions

PC paste specimens were immersed in room- and low-temperature seawater for 433 days. The specimen immersed in low-temperature seawater underwent more pronounced deterioration than that immersed in room-temperature seawater. The deteriorated specimen immersed in low-temperature seawater exhibited the following characteristics:

- The Ca dissolution front was deep within the sample, and portlandite dissolution was more pronounced than in the specimen immersed in room-temperature seawater;
- The porosity of the specimen increased with the dissolution of portlandite;
- On the collapsed mashy surface, C–(A-)S–H was decalcified and Mg-based hydrates (e.g., brucite and M–(A-)S–H) and thaumasite formed.

The increased solubility of portlandite at low temperatures led to significant Ca dissolution and subsequent deterioration via C–(A-)S–H decalcification and the formation of M–(A-)S–H and thaumasite, resulting in the weakening and collapse of the surface. Although few reports on the marine durability of cementitious materials have focused on the seawater temperature, it is necessary to consider this parameter when evaluating the durability of offshore and deep-sea infrastructure.

## Figures and Tables

**Table 1:** Previous studies of the marine deterioration of hardened cement materials.

Site	Zone	Seawater Temp (°C)	Time Period	Specimen
Kish Island, Iran [5]	Tidal zone	22–33 ^a^	up to 180 days	Paste and concrete, *w*/*b* ^c^ 0.2–0.4, mixed with SF and BFS
North coast of the Mediterranean Sea [8]	Splash zone	15–25 ^a^	4 to >60 years	Concrete, *w*/*c* ^c^ 0.34–0.65
Kurihama, Japan [4]	In seawater	15–28 ^a^	30 years	Concrete, *w*/*c* 0.52–0.55, with five different cements
Trondheim, Norway [1]	In seawater	5–15 ^a^	up to 30 years	Concrete, 2500 different mixes
Trondheim, Norway [7]	Tidal zone	5–15 ^a^	10 years	Concrete, *w*/*b* 0.4
Nine different locations in Norway and Denmark [9]	Mainly in seawater	minimum 5–15 ^a^	2–34 years	Concrete with 21 different mixes
Treat Island, USA [10]	In seawater	minimum −3 ^a^	24–25 years	Concrete, *w*/*b* 0.26–0.6, FA and SF containing lightweight blocks
Bandar-Abbas [6]	Tidal zone		3 years	Concrete, *w*/*c* 0.35, 0.40, 0.45, and 0.50, SF mixed with 5%, 7.5, 10, and 12.5%
Lab [3]	In seawater ^b^	20	560 days	Mortar, *w*/*b* 0.5, mixed with ground brick
Lab [2]	In seawater ^b^	20	33 weeks	Concrete, *w*/*c* 0.4

^a^ From Sea Temperature Info database [31]. ^b^ Seawater renewed every 28 days. ^c^ Water/binder ratio (*w*/*b*), water/cement ratio (*w*/*c*).

**Table 2:** Composition of Portland cement determined by X-ray fluorescence (unit: %).

SiO_2_	Al_2_O_3_	Fe_2_O_3_	CaO	MgO	SO_3_	Na_2_O	K_2_O	LOI ^a^
20.29	5.31	2.72	65.20	1.20	2.90	0.23	0.29	1.03

^a^ Loss on ignition.

**Table 3:** Paste mix proportions.

Component	Amount (g)
Portland cement (PC)	1200
Polycarboxylic ether (PCE)	9.6
Hydroxypropyl methylcellulose (HPMC)	5.8
Defoamer	2.4
Tap water	720

**Figure A1:**
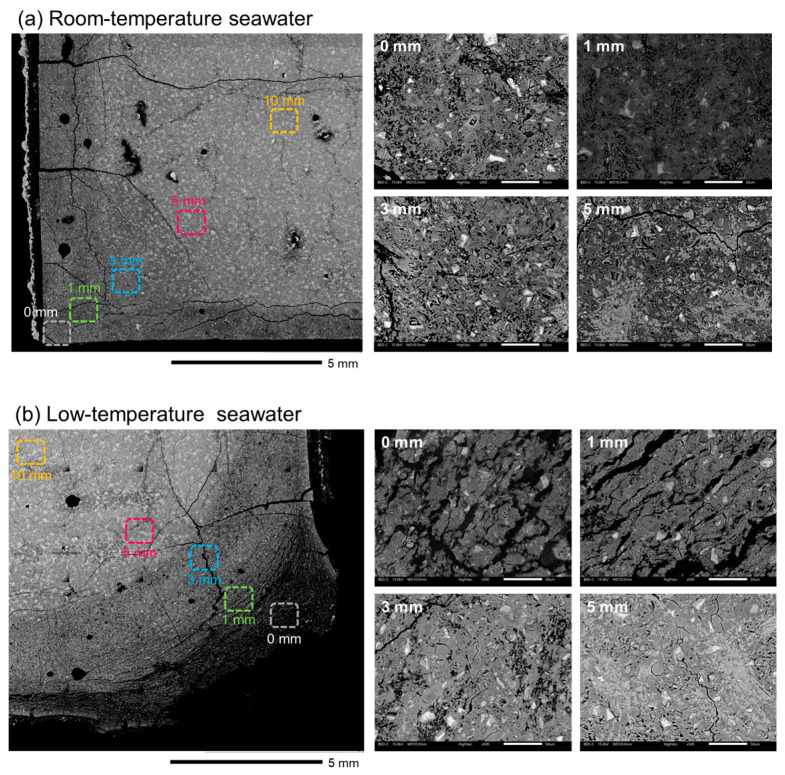
SEM montage images of the PC paste specimens immersed in (**a**) room-temperature seawater and (**b**) low-temperature seawater.

**Figure A2:**
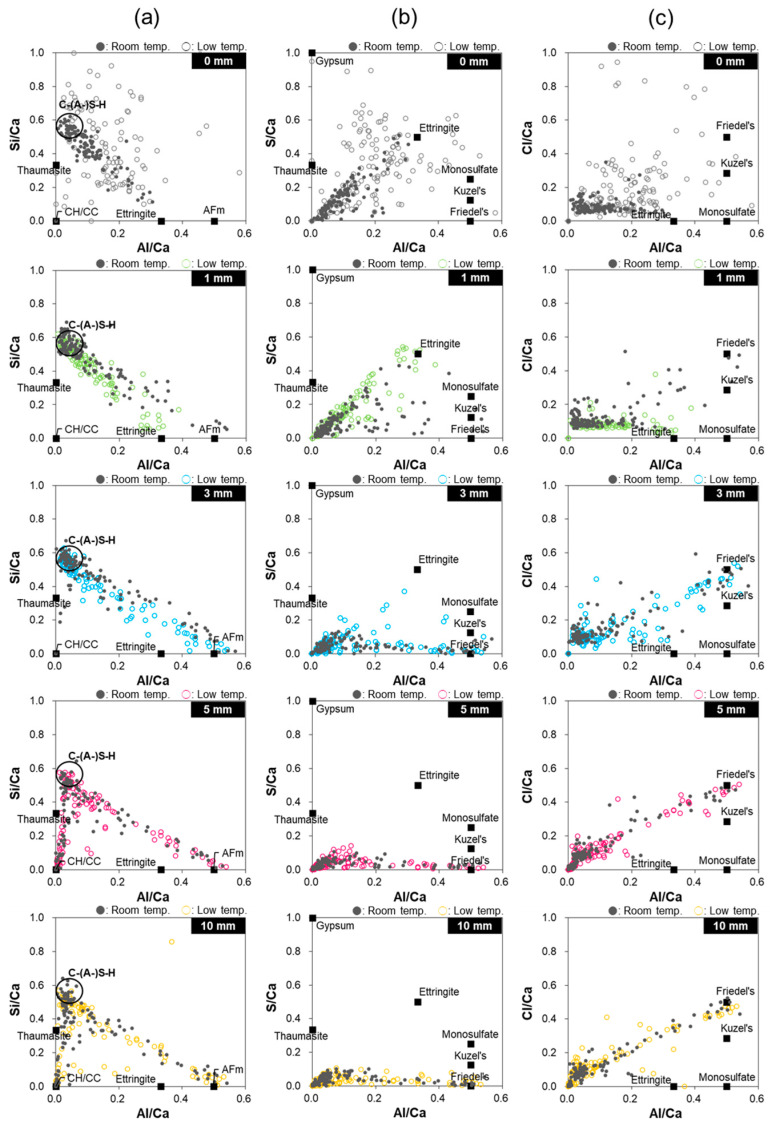
EDS point analysis at various depths for the specimens immersed in room- and low-temperature seawater shown in Figure A1: (**a**) Si/Ca, (**b**) S/Ca, and (**c**) Cl/C atomic ratios as functions of the Al/Ca atomic ratio.

## Data Availability

The data presented in this study are available in the article.

## Appendix A

Figure A1 shows the SEM montage images of the specimens immersed in room- and low-temperature seawater. The dotted squares in each montage image indicate where EDS point analysis was performed. Selected BSE images at depths of 0, 1, 3, and 5 mm are also shown. For the specimen immersed in low-temperature seawater, porous regions (black-colored areas in Figure 2b) are particularly abundant at depths of 0 and 1 mm.

## Appendix B

Figure A2 shows the EDS point analysis results at each depth for the specimens immersed in room- and low-temperature seawater shown in Figure A1. The Si/Ca, S/Ca, and Cl/Ca atomic ratios are plotted as functions of the Al/Ca atomic ratio.
